# Therapeutic Potential of Carbon Monoxide (CO) and Hydrogen Sulfide (H_2_S) in Hemolytic and Hemorrhagic Vascular Disorders—Interaction between the Heme Oxygenase and H_2_S-Producing Systems

**DOI:** 10.3390/ijms22010047

**Published:** 2020-12-23

**Authors:** Tamás Gáll, Dávid Pethő, Annamária Nagy, György Balla, József Balla

**Affiliations:** 1 Division of Nephrology, Department of Medicine, Faculty of Medicine, University of Debrecen, 4032 Debrecen, Hungary; gall.tamas@med.unideb.hu (T.G.); petho.david@med.unideb.hu (D.P.); nagy.annamari90@gmail.com (A.N.); 2 HAS-UD Vascular Biology and Myocardial Pathophysiology Research Group, Hungarian Academy of Sciences, University of Debrecen, 4032 Debrecen, Hungary; balla@med.unideb.hu; 3 Faculty of Medicine, University of Debrecen, Kálmán Laki Doctoral School, 4032 Debrecen, Hungary; 4 Department of Pediatrics, Faculty of Medicine, University of Debrecen, 4032 Debrecen, Hungary

**Keywords:** oxidized hemoglobin, heme, vascular disease, hemorrhage, hemolysis, heme oxygenase, carbon monoxide, carbon monoxide-releasing molecules, hydrogen sulfide

## Abstract

Over the past decades, substantial work has established that hemoglobin oxidation and heme release play a pivotal role in hemolytic/hemorrhagic disorders. Recent reports have shown that oxidized hemoglobins, globin-derived peptides, and heme trigger diverse biological responses, such as toll-like receptor 4 activation with inflammatory response, reprogramming of cellular metabolism, differentiation, stress, and even death. Here, we discuss these cellular responses with particular focus on their mechanisms that are linked to the pathological consequences of hemorrhage and hemolysis. In recent years, endogenous gasotransmitters, such as carbon monoxide (CO) and hydrogen sulfide (H_2_S), have gained a lot of interest in connection with various human pathologies. Thus, many CO and H_2_S-releasing molecules have been developed and applied in various human disorders, including hemolytic and hemorrhagic diseases. Here, we discuss our current understanding of oxidized hemoglobin and heme-induced cell and tissue damage with particular focus on inflammation, cellular metabolism and differentiation, and endoplasmic reticulum stress in hemolytic/hemorrhagic human diseases, and the potential beneficial role of CO and H_2_S in these pathologies. More detailed mechanistic insights into the complex pathology of hemolytic/hemorrhagic diseases through heme oxygenase-1/CO as well as H_2_S pathways would reveal new therapeutic approaches that can be exploited for clinical benefit.

## 1. Introduction

Heme (iron protoporphyrin IX) is the prosthetic group of proteins involved in diverse biological processes, such as mitochondrial respiration, oxygen-electron transport, and enzymatic reactions, making heme a fundamental of life. Later, it was discovered that heme is not only a prosthetic group of proteins but also the source of biologically active metabolic products produced by its complex elimination system in living organisms. This finding initiated the ‘heme story’ about 80 years ago. In 1945, Watson and co-workers showed that intravenous hematin is converted to bilirubin (BR) in humans [1]. Twenty years later, a nice paper demonstrated that the green pigment, biliverdin, is the direct product of the heme alpha-methenyl oxygenase enzyme [2]. The observation of Stocker was a milestone of heme metabolism research suggesting that BR possesses remarkable antioxidant activity in vitro [3]. The Maines’s group shed new light on the protective nature of the heme catabolic system in a brain ischemic model, where biliverdin reductase, through its fine regulation, balances the concentrations of biliverdin and neurotoxic BR [4].

In the second half of the 1980s, we have shown that free heme released from hemoproteins can be toxic to cells and organs and, moreover, to the whole organism. At the same time, we observed that an intracellular protective mechanism exists, the heme oxygenase-1 (HO-1)/ferritin system, preventing endothelial cell death caused by heme-catalyzed free radical injuries. In this heme sensitization model, ferritin but not HO-1 is the ultimate cytoprotectant [5]. In this way, we presented the first in vivo evidence that the induction of HO-1/ferritin synthesis is an endogenous, inducible, and protective system against heme stress, supporting Stocker’s hypothesis published in a review paper [6].

HOs exist in two isoforms; the inducible HO-1 is induced by variuos environmental stimuli, among them ultraviolet and radioactive irradiation, endotoxin, reactive oxygen stimuli, and of course, heme [7,8]. HO-2 is constitutively expressed; however, it is also induced by hypoxia [9]. In addition to its role in controlling the intracellular labile heme level [9], HO-2 is neuroprotective in cerebral ischemia [10], and mitigates transhemispheric diaschisis of the contralateral hemisphere in brain ischemia [11]. Besides, HO-2 gene polymorphism at an ATG start site is associated with Parkinson’s disease [12].

Solid evidence shows that heme toxicity is present in many human pathologies with hemolysis and hemorrhage [13]; this hypothesis is supported by the fact that both intra- and extracellular heme levels are finely regulated by multiple defense mechanisms. Extracellular free heme is rapidly scavenged by plasma hemopexin (Hpx) [14] and alpha-1-microglobulin, the latter of which is also present in most tissues, including the blood vessel walls [15,16]. Intracellular free heme leaking from hemoproteins is catabolized by heme oxygenases (HOs). However, severe hemolysis/hemorrhage rapidly overwhelms these extra- and intracellular protective systems, leading to cell, tissue, and organ damage.

Both carbon monoxide (CO) and hydrogen sulfide (H_2_S) were considered as potentially toxic gases; however, during the past decades, both of them have also been recognized as signaling molecules. CO is liberated during heme catabolism by HOs, which are the only currently known endogenous sources of CO. H_2_S is produced by enzymatic and non-enzymatic ways that will be discussed later in the paper.

In the present work, we aimed to summarize our current knowledge on how hemoglobin (Hb) and heme contribute to human pathologies with a special emphasis on the potential protective role of CO and H_2_S in hemorrhagic/hemolytic conditions.

## 2. Hemolysis- and Hemorrhage-Driven Damage Mechanisms

Hemolysis and hemorrhage are associated with many human pathologies, such as sepsis, brain hemorrhage, atherosclerosis with plaque rupture, sickle cell disease, hemolytic anemias, malaria, diabetic angiopathies, and mechanical injuries. The lysis of red blood cells (RBCs) liberates a large amount of cell-free Hb (Hb) into the bloodstream or tissues that is rapidly scavenged by haptoglobin (Hp) (reviewed by di Masi et al., [17]). Hb-Hp complexes are then taken up by macrophages via their CD163 receptors [18]. CD163 also mediates free Hb endocytosis in the absence of Hp [19]. Numerous studies have shown that oxidation of ferrous Hb by one or two (when Hb reacts with H_2_O_2_ or lipid hydroperoxides) electron-dependent steps forms metHb (Fe^3+^) and ferryl Hb (Fe^4+^ = O 2−) [20], the latter of which is reduced back to metHb with auto-reduction or reductants. Both MetHb and ferryl Hb are characterized by heme release, which induces extensive cell and tissue damage. Free heme is scavenged by the acute phase reactant protein hemopexin (Hpx) (reviewed by Montecinos et al. [21]) or alpha-1-microglobulin (A1M) [22]. Heme, taken up by cells, is catabolized by HOs releasing biliverdin, CO, and catalytically active iron, the latter of which is also a part of the pathology in hemolytic/hemorrhagic diseases.

Although hemorrhage is often followed by hemolysis, hemorrhage is not ultimately associated with hemolysis; Hb and heme-driven cell and tissue damage is strongly associated with RBC lysis. For example, after intracerebral hemorrhage, RBCs can be engulfed by macrophages/microglia called RBC efferocytosis, which may attenuate the adverse effect of free Hb/heme liberated during hemolysis [23]. In this natural protective process, both the phagocytosis-mediating scavenger receptor CD36 and nuclear factor erythroid 2–related factor 2 (Nrf-2), the master regulator of the cellular antioxidant system, among them HO-1, have an outstanding role [24,25,26].

### 2.1. Hb-Induced Toxicity

**Figure 1:**
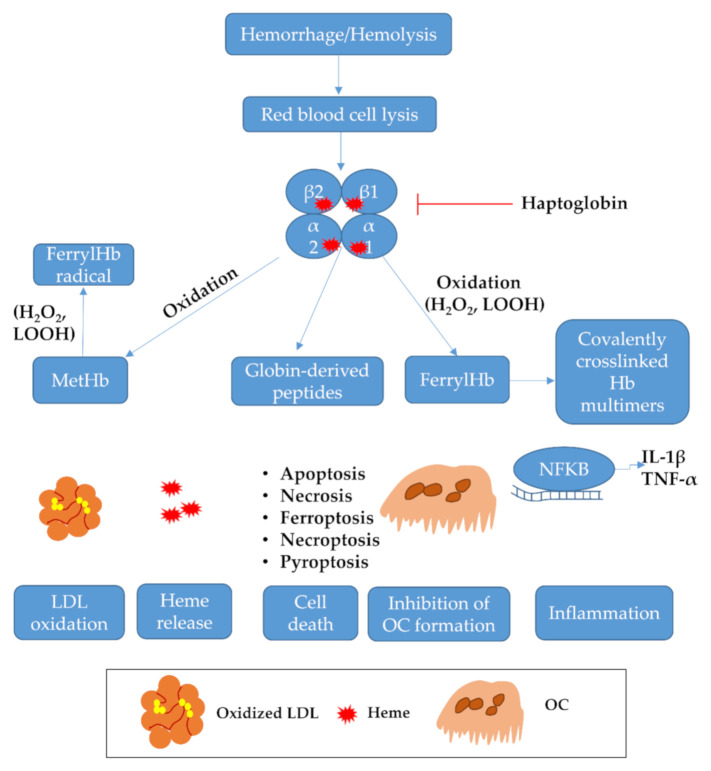
Hemoglobin-induced toxicity in hemolytic/hemorrhagic pathologies. Hemolysis and hemorrhage lead to the disruption of red blood cells with subsequent hemoglobin (Hb) release. Hb is rapidly scavenged by haptoglobin and taken up by macrophages via CD163 receptor. However, extensive hemolysis/hemorrhage rapidly overwhelms endogenous protective systems leading to Hb oxidation resulting in metHb, ferryl Hb, covalently crosslinked Hb multimers, or globin-derived peptides. Hb oxidation triggers low-density lipoprotein oxidation, cell death, inflammation, heme release, and inhibits oscteoclast formation. LDL: low-density lipoprotein; Hb: hemoglobin; NFΚB: nuclear factor kappa-light-chain-enhancer of activated B cells IL-1β: interleukin-1β; TNF-α: tumor necrosis factor-α; OC: osteoclast.

Hb oxidation plays a pivotal role in the pathology of hemolytic and hemorrhagic disorders by catalyzing low-density lipoprotein (LDL) oxidation as well as triggering inflammation and cell death. Recently, the βcysteine 93 residue of Hb has been shown as a gateway to its oxidative stability (reviewed by Alayash) [27]. As an iron compound, Hb is also a known Fenton-reagent facilitating hydroxyl-radical generation from activated oxygen species [28,29]. Interestingly, globin-derived peptides released by Hb oxidation also pose a threat to cell integrity; another exciting study has revealed that ferryl Hb directly interrupts osteoclastic differentiation of macrophages (Figure 1).

#### 2.1.1. Hb Triggers LDL Oxidation

LDL oxidation is a hallmark of vascular diseases [30,31]. This suggests that scavenging Hb may prevent the formation of oxidized LDL (oxLDL); this theory is supported by a recent study showing that in sickle cell disease patients, the absence of Hp is associated with increased lipid peroxidation and oxidized LDL deposition in the pulmonary artery [32], suggesting the key role of Hp to prevent free Hb-catalyzed extensive LDL oxidation (Figure 1). OxLDL triggers cell and tissue damage via multiple mechanisms, such as inflammation [33], inflammation-driven thrombosis [34], or cell death, that have been discussed by excellent reviews. In addition to its role in inflammation and cell death, oxLDL also facilitates the differentiation of a novel macrophage phenotype (Mox) that is markedly different from the traditional M1 (pro-inflammatory) and M2 (pro-resolving or anti-inflammatory) macrophages. Mox is characterized by decreased phagocytic/chemotactic activity and the abundant overexpression of Nrf2-mediated redox-regulatory genes. Mox develops both from M1 and M2 macrophages in response to oxidative tissue damage, representing a new macrophage phenotype involved in the pathogenesis of atherosclerosis [35].

#### 2.1.2. Hbs as Pro-Inflammatory Stimuli

Inflammation plays a central role in vascular diseases. In endothelial cells (ECs), ferryl Hb exerts a potent pro-inflammatory effect, leading to nuclear factor kappa-light-chain-enhancer of activated B cells (NFΚB) activation [36], and this requires actin polymerization and c-Jun N-terminal kinase as well as the p38 mitogen-activated protein kinase signaling (Figure 1). In ECs, ferryl Hb activates the NLR family pyrin domain-containing 3 (NLRP3) inflammasome with subsequent interleukin-1β (IL-1β) release [37]. Others have shown that ferryl Hb triggers active IL-1β production in LPS-primed macrophages as well as caspase-1 activation and IL-1β cleavage in mice [38]. The concept of hemorrhage-induced NLRP3 activation is also demonstrated in brain hemorrhage, where the suppression of NLRP3 by recombinant adenovirus mitigates inflammation and brain injury after intracerebral hemorrhage [39]. The pro-inflammatory potential of Hb is also underlined by a recent study, which has shown that Hb injected into the lateral brain ventricle provokes global inflammation of the brain [40].

Interestingly, several studies have shown that not only Hb oxidation but also its post-transcriptional modification might be associated with coronary artery disease. Glycosylated Hb predicts the severity of disease regardless of diabetic status [41]. Besides, oxLDL levels are correlated with glycosylated Hb levels in non-diabetic patients, suggesting a possible role of glycosylated Hb in cardiovascular diseases.

Overall, hard evidence supports that Hb is a potent pro-inflammatory stimulus to the cells in hemorrhagic/hemolytic diseases, and oxidized Hb is an important part of the pathology in these human disorders.

#### 2.1.3. Hemolysis-Induced Cell Death

Hemorrhage/hemolysis triggers Hb/heme-induced cell death, but the mechanism of this is still ill-lit (Figure 1). In their recent work, Yuan et al. showed a receptor-interacting protein-4 (RIP3)-induced necroptotic cell death in rat brain injury; besides, conditioned medium derived from Hb-activated microglia also triggers necroptosis in primary neurons [42]. In cultured neurons, inhibitors of necroptosis and ferroptosis but not apoptosis and autophagy protect cells from Hb and heme-induced cell death [43]. In contrast to this, others have shown that heme induces autophagic cell death in neurons [44]. To this end, the exact mechanism of neuronal cell death in response to hemorrhage is still unknown and needs to be further analyzed in upcoming studies.

Given that Hb is an iron-containing protein, it has the potential to induce ferroptosis. Ferroptosis is an iron-dependent form of cell death characterized by the accumulation of lipid reactive oxygen species [45]. Ferroptosis is characterized by decreased mitochondrial volume, reduced or disappeared mitochondrial cristae, and increased bilayer membrane density [45,46]. Biochemically, ferroptosis is strongly dependent on glutathione peroxidase 4 (GPX4) and ferroptosis suppressor protein 1 (FSP1). GPX4, which requires glutathione, breaks down lipid hydroperoxides protecting cells from ferroptosis [47]. FSP1 acting as oxidoreductase reduces ubiquinone (coenzyme Q10) to ubiquinol, a powerful lipophilic radical scavenger that antagonizes the accumulation of lipid ROS within membranes [48,49]. Others, among them heat shock protein beta-1 and Nrf-2, negatively, while others, such as NADPH oxidase and p53, positively regulate ferroptosis [50]. Given that catalytically active iron drives lipid peroxidation, ferroptosis is effectively inhibited by iron chelatros and inhibitors of lipid peroxidation [50].

In macrophages, heme-induced programmed cell death can be inhibited by necrostatin-1, a well-known inhibitor of necroptosis [51]. On the contrary, in A549 lung carcinoma cells, heme-induced cell death can be counteracted by neither necroptosis nor ferroptosis inhibitor [52]. In choroid plexus epithelial cells, hemorrhagic cerebrospinal fluid and metHb, as well as heme, display apoptotic and necrotic cell death [53]. A clinical study also suggests that apoptosis is a driving mechanism of cell death after intracerebral hemorrhage [54]. Besides, pyroptotic cell death represents another way of cell death after subarachnoid hemorrhage (SAH) [55]. Overall, these suggest that hemorrhage/hemolysis can induce cell death in a variety of ways, possibly in a cell type-specific manner that necessitates further research in this field.

#### 2.1.4. The Role of Globin-Derived PEPTIDES in Hb Toxicity

Hb oxidation also generates globin-derived peptides that are present in atherosclerotic lesions or after intraventricular hemorrhage in the brain [37]. Hb-derived peptides are also present in Alzheimer disease [56] and might be involved in various biological processes as bioactive signaling molecules [57]. Posta and co-workers showed that Hb-derived peptides do not bind to Hp or albumin, trigger endothelial damage, and facilitate monocyte adhesion to the endothelium (Figure 1). Besides, Hb-derived peptides induce tumor necrosis factor-α (TNF-α) expression and also facilitate NLRP3 inflammasome formation followed by IL-1β expression [37]. This suggests that not only oxidized Hb but also globin-derived peptides might be implicated in cell and tissue damage in atherosclerotic plaque and brain hemorrhage. This study highlights the importance of further research aimed at globin-derived peptides, since Hp, which detoxifies free Hb, is inefficient to scavenge these peptides.

#### 2.1.5. FerrylHb As an Inhibitor of Osteoclast Activity

In atherosclerotic plaques, calcium deposition is common representing an important part of the pathology. Evidence shows that osteoclast-like cells (OLCs) are present in calcified atherosclerotic plaques and might be implicated in mineral resorption of the arteries (Figure 1) [58,59]. A recent study has shown that ferryl Hb but not ferrous Hb disturbs the differentiation of OLCs from macrophages by directly interrupting the binding of receptor activator of nuclear factor-kappa-Β ligand (RANKL) to its receptor RANK, which is a key initiation step of OLC differentiation [60]. As a consequence of this, ferryl Hb blunts the bone resorption activity of OLCs. In human carotid artery specimens, OLCs are present in calcified atheromas, but their presence is significantly lower in hemorrhaged lesions that are characterized by ferryl Hb; this suggests that ferryl Hb impairs OLC formation in calcified atherosclerotic plaques. To this end, we postulate that oxidized Hb might also compromise the endogenous protective potential of vascular tissues, which further amplifies Hb-driven damage.

### 2.2. Heme-Induced Toxicity

Heme release after Hb oxidation [61] is likely to be involved in hemolytic/hemorrhagic disorders. Interestingly, the different Hb redox states—ferrous (Fe^2+^), ferric (Fe^3+^), and ferryl (Fe^4+^)—loose heme with different kinetics; ferric Hb releases heme at markedly higher rates than ferryl Hb [62]. Oxidants (H_2_O_2_, nitrite, peroxynitrite, and hypochlorous acid) formed during inflammation differently induce heme loss from Hb and hemolysates. H_2_O_2_ is the most potent inducer of heme loss, suggesting that heme loss is possibly triggered by continuous generation of H_2_O_2_ rather than other oxidants.

**Figure 2:**
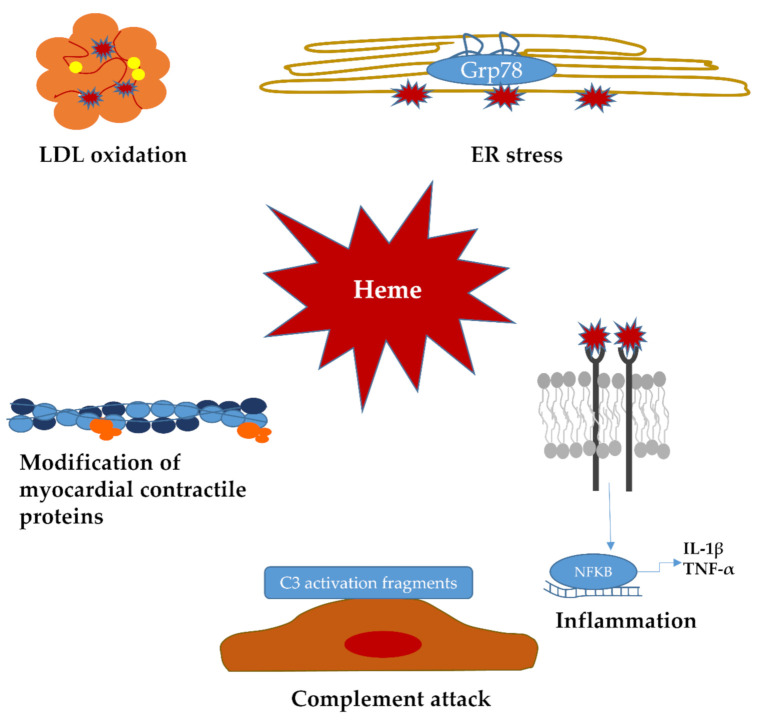
Mechanisms of heme-induced toxicity. Oxidation of hemoglobin results in heme release that triggers a wide range of cell damages. Heme intercalates into low-density lipoprotein (LDL) particles and amplifies LDL oxidation. Free heme induces the oxidative modification of myocardial contractile proteins and triggers endoplasmic reticulum stress in a variety of cells. Heme also mediates complement attack against the endothelium and possesses a potential pro-inflammatory effect. LDL: low-density lipoprotein; NFΚB: nuclear factor kappa-light-chain-enhancer of activated B cells; IL-1β: interleukin-1β; TNF-α: tumor necrosis factor-α; Grp78: glucose-regulated protein 78; ER: endoplasmic reticulum.

To date, numerous studies have demonstrated the toxic effects of heme, which contributes to the pathology of hemolytic diseases. From an evolutionary aspect, multiple mechanisms have been evolved to detoxify free heme; such mechanisms exist in prokaryotic organisms (reviewed by Anzaldi et al., [63]). Hemozoin, an insoluble crystalline of free heme, is essential to survive in hematophagous organisms, such as malaria parasites. In Vertebrates, free heme is specifically scavenged by extracellular heme-binding protein hemopexin, and alpha-1-microglobulin, or, by serum albumin [64]. Besides, HOs also protect cells from heme toxicity by catabolizing heme into iron, biliverdin, and CO. In this chapter, we will discuss recent findings in the field of heme-driven damages with a special emphasis on LDL oxidation, inflammation, and endoplasmic reticulum (ER) stress, all of which are implicated in hemolytic/hemorrhagic pathologies (Figure 2).

#### 2.2.1. Heme-Induced LIPID Modifications

Heme catalyzes the oxidation of lipids [65], and LDL [66] that has a crucial role in atherosclerosis [67]. Heme intercalates into LDL particles and facilitates their oxidative modifications (Figure 2) that are amplified by H_2_O_2_ followed by catalytically active iron release, which further boosts oxidative damage [66]. Hemopexin prevents not only heme-induced modification of LDL but also its Hb-induced peroxidation [68]. Others have shown that LDL oxidation is induced by a heme-initiated globin radical [30], and the target site for LDL oxidation is near the hydrophobic core of the lipoprotein [69].

OxLDL triggers a broad range of cellular damages, among them inflammation and endoplasmic reticulum (ER) stress. Evidence shows that oxLDL-driven cell death in vascular endothelial cells is mainly mediated by ER stress via the RNA-dependent protein kinase (PKR)-like ER kinase (PERK)/C/EBP-homologous protein (CHOP) pathway [70]. ER stress is also implicated in the control of the lipid metabolism of macrophages by increasing lipid uptake but decreasing lipid efflux. OxLDL-induced lipid uptake is boosted by ER stress inducer while it is attenuated by ER stress inhibitor [71]. Interestingly, CD36-mediated oxLDL uptake triggers ER stress, which, in turn, upregulates CD36 expression by a vicious circle mechanism. Disturbed lipid metabolism of macrophages is, at least partly, linked to ER stress by CHOP signaling [72].

NLRP3 activation-driven inflammation has a crucial role in oxLDL-induced cell damage [73,74]. NLRP3 depletion decreased apoptotic cell death, reduced ROS generation, and preserved proliferative potential in human aortic ECs challenged by oxLDL [75]. Besides, EC-specific NLRP3-depletion markedly reduced the severity of atherosclerosis in ApoE-deficient mice on a high-fat diet. Overall, these results suggest that Hb and heme can indirectly drive atherosclerosis by inducing inflammation and ER stress via the oxidative modification of LDL.

#### 2.2.2. Heme-Induced Protein Modifications

Heme is reported to modify not only LDL but also other proteins. In the presence of reducing agent and oxygen, heme induced the oxidative degradation of myoglobin [76]. Heme also alters myocardial contractility via post-translational modification of contractile proteins and binding to myosin light-chain 1 in human cardiomyocytes (Figure 2) [77]. This raises the notion the heme-induced protein modifications might also be involved in hemorrhagic/hemolytic diseases, which will be discussed later in this work.

#### 2.2.3. Heme-Induced ER Stress

Protein misfolding and ER stress are involved in many human pathologies [78]. Having established that heme induces the oxidative modification of LDL and other proteins, it is plausible that heme may target other proteins, leading to their misfolding and subsequent ER stress. This hypothesis has been tested in a recent work, which has revealed that heme induces ER stress in human aortic smooth muscle cell culture, which is inhibited by the heme scavenger Hpx and A1M (Figure 2) [79]. Besides, hemorrhaged carotid plaques derived from patients who underwent carotid endarterectomy contained a significant amount of oxidized Hb and heme with the parallel activation of ER stress pathways, suggesting that hemorrhage/heme-induced ER stress might be involved in the pathology of atherosclerosis and hemorrhagic/hemolytic diseases. This notion is also supported by more recent studies. In mice, intravascular hemolysis promotes acute kidney injury with concomitant oxidative and ER stress [80]. In neurons, heme induces autophagic cell death via ER stress [44]. Overall, this suggests that heme-induced ER stress might be implicated in the pathogenesis of hemorrhagic/hemolytic disorders, which necessitates further research in this field.

#### 2.2.4. Heme As a Pro-Inflammatory Stimulus

Toll-like receptor 4 (TLR4) is a member of the toll-like receptor family that recognizes pathogens and plays an essential role in host defense [81]. TLR4 is activated by the bacterial cell wall component lipopolysaccharides (LPS) followed by pro-inflammatory cytokine production [82]. Many endogenous TLR4 ligands have been identified [83], including heme. Heme-induced TLR4 activation results in complex downstream signalization (Figure 2). Heme-induced TLR4 signaling results in EC activation and vasoocclusion in sickle cell disease (SCD) mice [84]. TLR4 activation also regulates labile heme pools by influencing BACH1 and HO-1 expression in macrophages in a species-specific manner [85]. In murine bone marrow-derived macrophages (BMDMs), LPS raises the labile heme level, represses BACH1, and induces HO-1, while in human BMDMs, it has the opposite effect, suggesting that TLR4 stimulation alters labile heme levels that plays an essential role in the BACH-1-mediated HO-1 level.

In a spinal cord injury model, heme induces TNF and TLR4 expression both in primary microglial cell culture and in mice. Preventing TLR4/TNF induction by tranexamic acid is accompanied by a reduction of cell death and improves the functional recovery of mice [86].

TLR4 is also implicated in heme-mediated microglia activation followed by NFKB activation, increasing pro-inflammatory cytokine expression and inflammatory injury after intracerebral hemorrhage [87]. TLR4-induced inflammation after intracerebral hemorrhage (ICH) stimulates neuronal apoptosis, which is decreased by IL-1β and TNF-α antagonists, while TLR4 knockout markedly increases the survival rate after ICH [88]. Heme also triggers NLRP3 inflammasome activation in ECs [89]. Furthermore, heme-activated TLR4 signaling is implicated in P-selectin-driven complement attack against the endothelium in a phenylhydrazine-induced hemolysis model [90]. In the murine model of trauma hemorrhage followed by resuscitation with stored blood, increased bacterial infection susceptibility and severity are associated with free heme in a TLR4-dependent manner as scavenging heme by Hpx or deletion of TLR4 prevents mortality [91]. Heme induces sickle pain in mice via TLR4-mediated ER stress and ROS [92]. Importantly, heme can trigger TLR4 activation with the subsequent inflammatory response through oxLDL [88].

In conclusion, heme triggers a wide range of damages via TLR4 signaling, which makes TLR4 a potential candidate for future therapeutic approaches in hemolytic/hemorrhagic diseases.

## 3. Carbon Monoxide

Exogenous carbon monoxide (CO) is generally regarded as a poisonous gas by forming carboxy-Hb that blocks Hb’s oxygen binding site; moreover, this prevents oxygen release from Hb at the capillary region of the circulation [93]. Other heme proteins are also the targets of CO, such as myoglobin, cytochrome c oxidase of the mitochondrial respiratory chain, or cytochrome p450-dependent monooxygenases. Inhibition of cytochrome c oxidase can lead to the generation of reactive oxygen species (ROS).

Intracellularly, CO is produced by Hos, suggesting its potential important role as an endogenous gasotransmitter. CO is one of the end-products of heme catabolism by heme oxygenase-1 (HO-1) and heme oxygenase-2 (HO-2). HO-1 is the only currently known inducible heme-catabolizing enzyme activated at the transcriptional level by a variety of stress stimuli, most importantly, by heme [7,94], while HO-2 is constitutively expressed. HOs convert heme into biliverdin, CO, and iron. In the past decades, CO has gained a lot of interest not only by its potential toxicity but also its remarkable cytoprotectant properties against inflammation [95,96], acute lung injury, atherosclerosis [97], or in organ transplantation [98]. This fueled the development of suitable CO delivery systems, such as CO-releasing molecules (CORMs). Importantly, not only CO but also BR converted from biliverdin by biliverdin reductase has remarkable protective properties; BR mitigates monocyte migration through endothelial cells and inhibits plaque formation in LDL receptor-deficient mice [99]. A decreased serum level of BR is an independent predictor of sublicinal atherosclerosis [100]. On the contrary, BR neurotoxicity is well-known, especially in newborns [101]. The remarkable role of BR in the human biology has recently been summarized by an elegant review article [102].

### 3.1. CO in Hemorrhage-Triggered Cell Death

CO has a prominent role to mitigate oxLDL-induced cell damage. In ECs, CORM-2 markedly reduces cell death and ROS formation triggered by oxLDL via improving mitochondrial function and blocking the Wnt/β-catenin pathway [103]. CORM-3 ameliorates the emotional deficits and neuronal cell death induced in the amygdala in a post-traumatic brain injury and hemorrhage shock and resuscitation (HSR) rat model by protein kinase G-ERK1/2 signaling [104]. CORM-3 also alleviates neuronal pyroptosis and improves neurological recovery in HSR through mitochondrial regulation mediated by the soluble guanylyl cyclase-cGMP pathway. Thus, CO administration could be a promising therapeutic strategy for hemorrhagic shock. In brain hemorrhage, CORM-3 attenuates neuronal pyroptosis and improves neurological recovery [105]. These findings support the notion that CO can ameliorate cell death in hemorrhagic/hemolytic pathologies via multiple mechanisms.

### 3.2. CO in Hemorrhage-Induced Inflammation

CO and CORMs possess remarkable anti-inflammatory properties [106] that implicate their use to ameliorate inflammation in diverse human pathologies. HBI-002, a liquid CO formulation administered orally, effectively decreases inflammatory response in SCD mice [84]. CORM-3 also attenuates lung injury triggered by TNF-α, inducible nitric oxide synthase, and IL-1β in an HSR-induced animal model [107]. Besides, CORM-3 enhances the expression of interleukin-10, one of the most significant anti-inflammatory cytokines in this model. Inhalation of CO gas also ameliorates hemorrhagic shock-induced lung injury by increasing peroxisome proliferator-activated receptor (PPAR)-γ expression, an anti-inflammatory transcriptional regulator in the lung [108]. The anti-inflammatory effect of CO is also demonstrated in other models. Inhaled CO decreased LPS-induced circulating pro-inflammatory cytokine level, and induced anti-inflammatory IL-10 through the mitogen-activated protein kinase, particularly the p38 pathway [109]. Others have found that the antiinflammatory effect of CO also involves the c-Jun N-terminal kinase (JNK) pathway [110] and heat shock factor 1 activities [111].

### 3.3. CO-Induced Cellular Metabolic Changes

**Figure 3:**
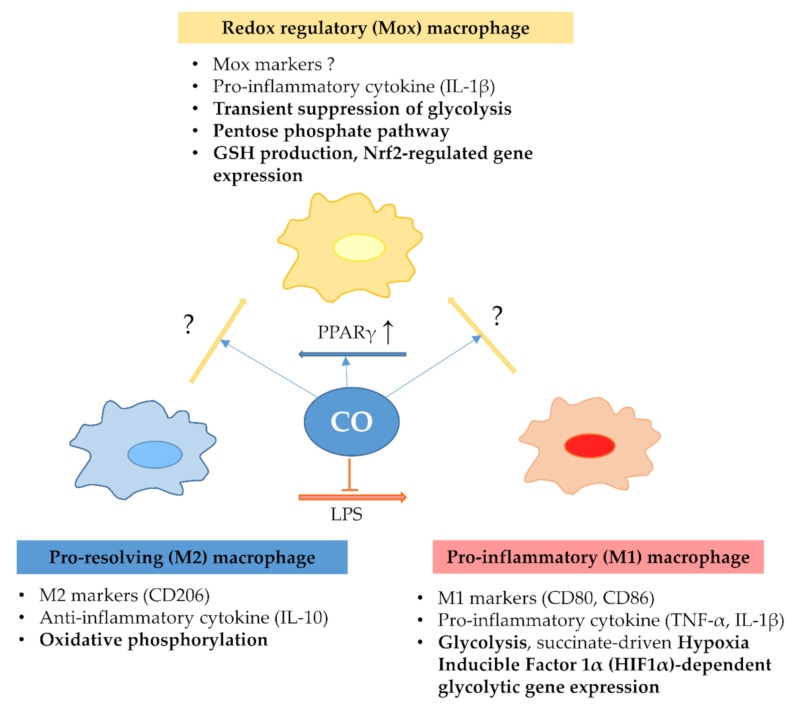
Carbon monoxide-induced phenotypic switch of macrophages. Carbon monoxide (CO), released during heme catabolism or CO-releasing molecules (CORMs), modulates macrophage matobolism and phenotype. Pro-resolving (M2) macrophages are dependnet on oxidative phosphorylation, while pro-inflammatory (M1) macrophages rely on glycolysis (Warburg effect) and hypoxia-inducible factor-1α-dependent glyolytic gene expressions, supporting inflammatory cytokine expressions. Mox macrophages, which are formed in atherosclerotic plaques by oxidized LDL, are characterized by the transient repression of glycolysis and inflammatory cytokine expression; however, these cells metabolize glucose via the pentose phosphate pathway (PPP), supporting their increased gluathion synthesis and nuclear factor erythroid 2-related factor 2 (Nrf2)-related antioxidant gene expression. Intrestingly, CO inhibits the phenotypic switch towards the pro-inflammatory phenotype triggered by lipopolysaccharide (LPS) and facilitates pro-resolving macrophage formation by inducing peroxisome proliferator-activated receptor gamma (PPARγ). Recently, the heme oxygenase/CO pathway has been suggested to activate PPP, which might be implicated in heme detoxication by macrophages. IL-1β: interleukin-1β; GSH: glutathione; IL-10: interleukin-10; TNF-α: tumor necrosis factor-α; PPARγ: peroxisome proliferator-activated receptor-γ; LPS: lipopolysaccharide; ?: currently unrevealed mediators; ↑: increase.

Heme as well as the heme catabolism by-product iron have been reported to trigger macrophage phenotypic switch toward an M1 pro-inflammatory phenotype, which is inhibited by the heme scavenger Hpx in a murine model of SCD [112]. The plasticity of macrophage function is suggested to be linked to their cellular energy metabolism (Figure 3).

In general, pro-inflammatory (M1) macrophages are characterized by glycolysis and succinate-driven hypoxia-inducible factor 1α (HIF1α)-dependent glycolytic gene expressions that are necessary for pro-inflammatory cytokine production [113]. In contrast, anti-inflammatory (M2) macrophages rely on oxidative phosphorylation [114]. CORM-3 has been reported to modulate the M1/M2 phenotype ratio in alveolar macrophage cell culture, suggesting that CO can modulate M1/M2 balance [115]. Similar to macrophages, microglia respond to inflammatory stimuli by a metabolic switch from oxidative phosphorylation to glycolysis, which supports their inflammatory response releasing IL-6, IL-1β, and TNF-α. The M2 phenotype characterized by oxidative metabolism is implicated in the resolution of inflammation and tissue repair [116]. However, recent work has shown that CO has the potential to reverse metabolic changes to attenuate the inflammatory response triggered by LPS [117]. CORM-3 also reduces glycolysis-dependent NLRP3 inflammasome activation in response to LPS [118]. However, others have shown that CO triggers mitochondria-derived ROS production in macrophages, promoting HIF1α activation and stabilization followed by the expression of transforming growth factor-β (TGF-β) that is involved in cytoprotective preconditioning [119]. In ECs, CO released by CORM—401 uncouples mitochondrial respiration and inhibits glycolysis [120], increases nitric oxide (NO) synthesis as well as glucose flux towards the pentose phosphate cycle [121]. In their exciting work, Bories and co-workers showed that heme drives a metabolic switch in macrophages from oxidative phosphorylation towards the pentose phosphate cycle, which is controlled by the HO-1-dependent CO generation [122]. This metabolic switch highly resembles the metabolic reprogramming of M1 and M2 macrophages during the differentiation of these cells from the Mox phenotype in response to oxLDL. Mox macrophages are strongly dependent on glucose metabolism and the pentose phosphate pathway to fuel glutathione (GSH) production and the Nrf2-dependent antioxidant pathway, both of which require NADPH as a reducing equivalent produced by the pentose phosphate pathway [123].

Overall, CO induces the metabolic reprogramming in multiple cell types that might be implicated in the CO-mediated protective effects in hemolytic/hemorrhagic diseases that need to be further investigated in future studies.

## 4. Hydrogen Sulfide

Hydrogen sulfide (H_2_S) used to be considered a toxic gas [124]; however, it is present in the brain at a quite high concentration, which raises its possible role as an endogenous neuromodulator [125]. H_2_S production is also well known in bacteria in which it is associated as a defense mechanism against oxidative stress provoked by antibiotics [126]. H_2_S regulates diverse reactions, such as neuronal synaptic transmission, inflammation, angiogenesis, and vascular myogenic tone.

Endogenous H_2_S is produced enzymatically as well as non-enzymatically in humans. Enzymatic H_2_S production involves cystathionine β-synthase (CBS) and cystathionine γ-lyase (CSE), both of which produce H_2_S mainly from L-cysteine [127]. The third currently known H_2_S-producing enzyme is 3-mercaptopyruvate sulfurtransferase (3-MST), which produces H_2_S from 3-mercaptopyruvate, which is synthesized from L-cysteine and α-ketoglutarate by cysteine aminotransferase [128]. Recently, the interesting work of Yang et al. revealed that H_2_S production from cysteine is catalyzed by iron and vitamin B6 [129]. The importance of this study is that in hemolytic/hemorrhagic diseases, a high amount of H_2_S might be released due to the remarkable catalytic potential of iron liberated during heme catabolism. In the case of non-enzymatic H_2_S production, H_2_S can derive from thiosulfate [130] or sulfur-containing molecules found in herbs, such as garlic [131].

The possible beneficial role of H_2_S in vascular diseases has been raised by Wang et al., showing that the H_2_S donor NaHS inhibits atherosclerotic plaque formation in ApoE-knockout mice, while inhibition of CSE activity by DL-propargyl glycine accelerates atheroma progression [132]. These findings raise the hypothesis that H_2_S donors might be valuable tools in the armamentarium of anti-atherosclerotic agents.

Others have also shown that intracerebral hemorrhage impairs endogenous H_2_S production possibly by reducing CBS; however, NaHS or S-adenosyl-L-methionine, a specific CBS agonist, restores brain and plasma H_2_S levels and counteracts neurological deficits as well as the inflammatory response triggered by hemorrhage [133]. Interestingly, not only H_2_S donors but also L-cysteine, which is a precursor of H_2_S generated by CBS, reduces inflammation, ROS generation, ER stress, and HO-1 expression after SAH in rats [134]. These results support the hypothesis that H_2_S might be a potential protective stratagem to mitigate hemorrhagic/hemolytic diseases. In this chapter, we will discuss the potential protective role of H_2_S in hemorrhagic/hemolytic diseases primarily focusing on its role in LDL and Hb oxidation, cellular injury, and inflammation.

### 4.1. H_2_S as an Inhibitor of Atherosclerosis and Hb Oxidation

The protective effect of H_2_S and the endogenous H_2_S -producing system is supported by several lines of evidence. Importantly, oxLDL has a direct adverse effect on H_2_S production by downregulating the CSE/H_2_S pathway, while overexpression of CSE reduces oxLDL-driven TNF-α expression in macrophages [135]. Besides, oxLDL induces the hypermethylation of the CSE promoter, decreasing its expression in macrophages [136]. In addition to this, oxLDL also downregulates the CBS/H_2_S pathway both in human and murine macrophages followed by NFKB p65 phosphorylation that is inhibited by exogenous H_2_S [137]. In addition, H_2_S eliminates lipid hydroperoxides in oxLDL and inhibits HO-1 induction by oxLDL in ECs [138]. Conclusively, H_2_S derived from either endogenous or exogenous sources possesses an important protective effect against oxLDL-driven damages in hemolytic/hemorrhagic pathologies.

**Figure 4:**
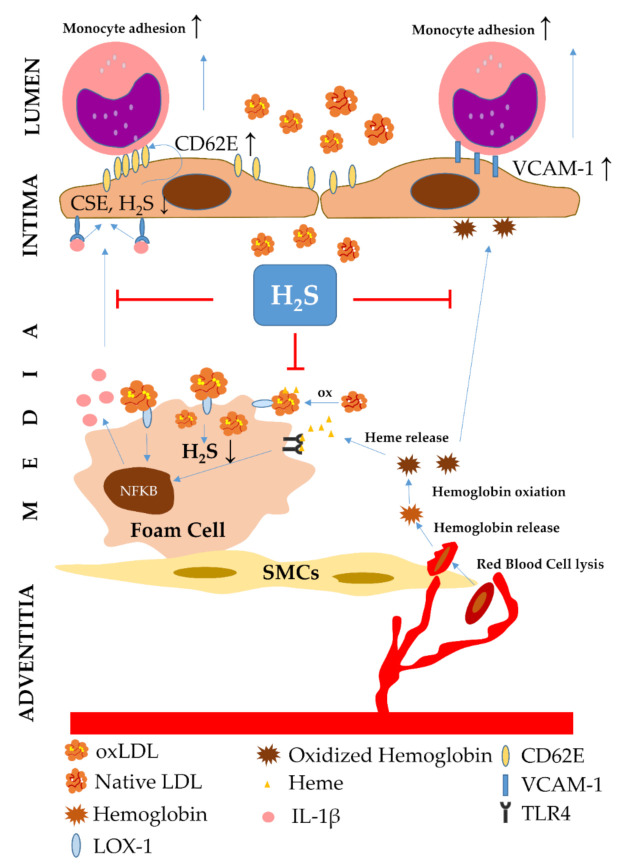
Protective effect of H_2_S on hemoglobin-driven damages in atherosclerosis. Neovascularization, budding of tiny blood vessels from vasa vasorum, is common during atherosclerotic plaque progression due to the thickening of the vessel wall in the plaque area. However, neovascularized capillaries are immature, leading to blood leakage into the great vessel wall. Red blood cells invading the highly oxidative environment of plaques lyze followed by the release and oxidaTable 1. (IL-1β). IL-1β downregulates cystathionine γ-lyase (CSE) activity and hydrogen-sulfide (H_2_S) production in endothelial cells, which induces CD62E expression, leading to monocyte adhesion to the endothelium. Oxidzed Hb also triggers the expression of adhesion molecules, such as vascular cell adhesion protein-1 (VCAM-1), that facilitates monocyte adhesion to the endothelium and their trans-endothelial migration. H_2_S inhibits oxidized LDL-induced inflammation and CD62E expression as well as oxidzed Hb-induced VCAM-1 expression in endothelial cells, inhibiting monocyte adhesion and trans-endothelial migration together with subsequent foam cell formation. CSE: cystathionine γ-lyase; VCAM-1: vascular cell adhesion protein-1; NFΚB: nuclear factor kappa-light-chain-enhancer of activated B cells; SMC: smooth muscle cell; oxLDL: oxidized low-density lipoprotein; IL-1β: interleukin-1β; TLR4: toll-like receptor 4; LOX-1: lectin-like oxidized low-density lipoprotein receptor-1; ↑: increase; ↓: decrease.

As discussed above, Hb oxidation significantly contributes to the pathomechanism of atherosclerosis (Figure 4).

Theoretically, inhibition of Hb oxidation may have a beneficial role in atherosclerosis. To support this, a recent study has shown that H_2_S significantly mitigates Hb oxidation, preventing the formation of ferryl Hb derivatives and Hb–lipid interactions. H_2_S also lowers the expressions of adhesion molecules triggered by oxidized Hb in ECs, thereby preserving endothelium integrity [139].

A more recent study has revealed that EC-specific deletion of CSE elevates CD62E expression with subsequently increased monocyte adherence to the endothelium in mice [140]. Although CSE expression is upregulated both in mice and human atheromas, H_2_S was decreased inside of atheromas as well as in the circulation, possibly due to an IL-1β-driven inhibition of CSE enzyme activity. The authors showed that CSE-derived H_2_S abolished the RNA binding protein human antigen R (RBPA R) homodimerization and subsequent CD62E induction by S-sulfhydration of RBPA R at Cys13; however, this beneficial effect of CSE-derived H_2_S was lost as a consequence of the inhibition of CSE enzyme activity by vascular inflammation. These studies raise the important notion that the CSE/H_2_S system represents an atheroprotective pathway for removing or limiting the formation of oxidized Hb and lipid derivatives in atherosclerotic plaques.

### 4.2. H_2_S in Hemorrhage-Induced Cell Injury

Free Hb and heme liberated from red blood cells during hemorrhage/hemolysis trigger injuries of the neighboring innocent cells. Available data suggest that H_2_S might be protective against Hb/heme-driven cell death. In an SAH rat model, NaHS decreased apoptotic cell death. Interestingly, the neuroprotective effect of NaHS was dependent on the L-type Ca^2+^ channel, since the Ca^2+^ channel agonist nifedipine decreased the protective effect of NaHS [141]. In ICH, endogenous H_2_S production is low due to the decrease in CBS expression, which is the predominant H_2_S-producing enzyme in the brain [142]. However, NaHS could restore the expression of CBS and H_2_S production and reduce cellular apoptosis as well as autophagy. Interestingly, the CBS inhibitor aminooxy acetic acid counteracted the beneficial effects of NaHS, suggesting the pivotal protective role of CBS.

Evidence suggests that H_2_S might ameliorate hemorrhage-induced cell death in the brain. In cultured neurons, inhibitors of necroptosis and ferroptosis protected cells from Hb and heme-induced cell death [43]. Iron toxicity and ferroptotic cell death are implicated in brain hemorrhage [143]. Recently, CBS has been suggested as a new negative regulator of ferroptotic cell death [144]; moreover, exogenous H_2_S inhibits necroptosis in ECs triggered by a high glucose concentration [145]. These data strengthen the idea that H_2_S may protect against ferroptosis and necroptosis in the case of hemorrhagic diseases.

### 4.3. H_2_S, Inflammation, and TLR4 Signaling in Hemorrhagic/Hemolytic Pathologies

H_2_S acts as a potent anti-inflammatory molecule in several cell types via inhibition of the TLR4/NFΚB pathway PMID: 31,153,885 as well as NLRP3 inflammasome formation [146]. In a murine model of atherosclerosis, NaHS improved plaque stability by increasing the collagen content of vascular tissues. Besides, H_2_S decreased apoptosis marker expressions as well as the mRNA expression of the oxLDL scavenger receptor, the lectin-like oxidized low-density lipoprotein (LDL) receptor-1 (LOX-1), in response to oxLDL in vascular smooth muscle cultures [147]. In a murine model of atherosclerosis, the H_2_S donor GYY4137 decreased plaque area and the volume of foam cells and ameliorated the pro-inflammatory cytokine level possibly by reducing TLR4 expression [148].

Diabetes mellitus is an important risk factor for accelerated atherosclerosis [149]. In a diabetes-accelerated atherosclerotic model, the H_2_S donor GYY4137 reduced plaque formation, NLRP3 inflammasome activation, and the expression of both intercellular cell adhesion molecule 1 (ICAM1) and vascular cell adhesion molecule 1 (VCAM1) [150]. H_2_S-mediated protein sulfhydrylation of Keap1 at Cys151 also results in the nuclear translocation of Nrf2 followed by HO-1 expression and decreased oxidative stress that mitigates diabetes-accelerated atherosclerosis in mice [151]. In addition, H_2_S inhibits NFKB p65 activation triggered by oxLDL via S-sulfhydration of Cys38 of NFKB [137] as well as NLRP3 inflammasome activation by reacting with the Cys269 of c-Jun kinase in macrophages [152].

The anti-inflammatory potential of H_2_S is also suggested in brain hemorrhage. In a rat model of SAH, the H_2_S donor NaHS significantly reduced neuroinflammation and cognitive impairment [153]. In experimental cerebral malaria characterized by blood leakage into the brain, CBS expression and H_2_S bioavailability are low, which is associated with the damage of the blood-brain barrier, suggesting that a low brain H_2_S level may contribute to the pathology of experimental cerebral malaria [154].

## 5. The Interplay between the H_2_S and HO-1/CO System

**Figure 5:**
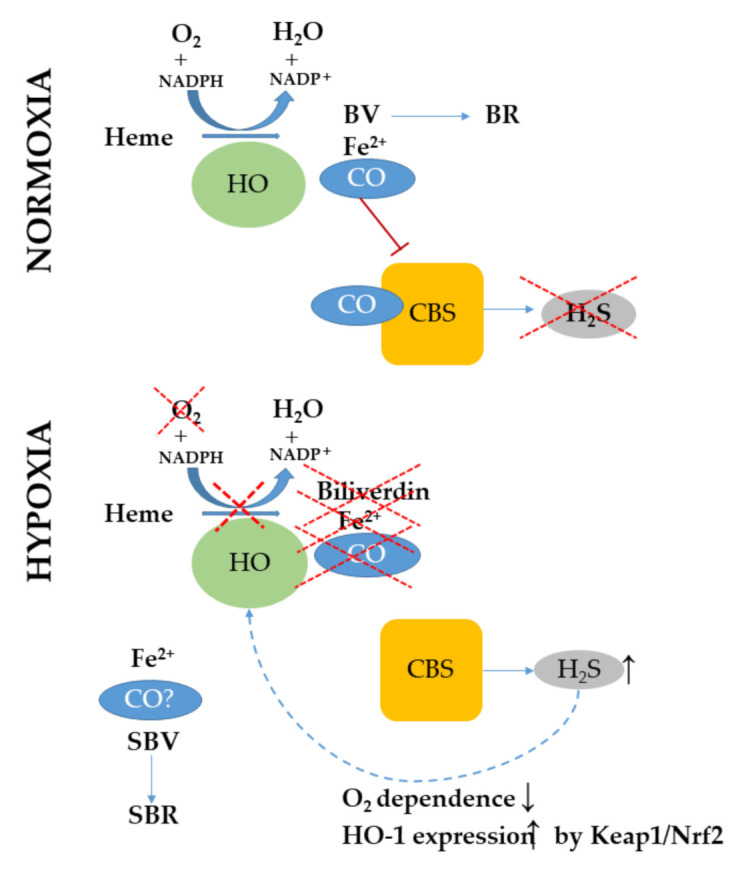
Interaction between the heme oxygenase/carbon monoxide and cystathionine beta synthase/H_2_S systems in vascular pathologies. In a normoxic environment, sufficient oxygen supply enables heme degradation by heme oxygenases (HOs) forming iron, biliverdin (BV), and carbon monoxide (CO), the latter of which inhibits cystathionine β-synthase (CBS) activity, leading to decreased hydrogen sulfide (H_2_S) production. BV is then formed to bilirubin (BR) by biliverdin reductase. In hypoxia, heme catabolism and CO generation by HOs is disturbed due to the limited oxygen availability, resulting in the derepression of CBS activity with concomittant H_2_S synthesis. H_2_S decreases the oxygen dependence of HOs, which enables heme catabolism leading to iron and sulfo-BV formation, the latter of which is converted to sulfo-BR. It is currently unknown whether CO is formed during this reaction. Besides, H_2_S might also increase HO-1 expression via the sulfenylation of Keap-1 that releases Nrf2, which induces Nrf2-regulated gene expression, including HO-1. BV: biliverdin; BR: bilirubin; CO: carbon monoxide; CBS: cystathionine β-synthase; HO: heme oxygenase; Keap-1: Kelch-like ECH-associated protein 1; Nrf2: nuclear factor erythroid 2-related factor 2; SBV: sulfur-containing biliverdin; SBR: sulfur-containing bilirubin; ↑: increase; ↓: decrease; red X: inhibition.

The human CBS enzyme is a pyridoxal-5′phosphate-dependent heme protein [155]. Although heme is not essential for CBS activity, the hemeless mutant displays 40% of wildtype enzyme activity [156]. Importantly, the heme moiety represents a potential regulatory point of CBS activity. CBS activity is almost doubled when ferrous heme is oxidized to the ferric state [157]. CO can bind to CBS, leading to its inactivation [158]. In hypoxia, cerebral vasodilation emerges from the interplay between the HO-2/CO and CBS/H_2_S system [159]. CO is constitutively generated by HO-2 in the brain, which inhibits CBS and H_2_S generation. Hypoxia-induced arteriolar vasodilation in the brain also regulated via the interaction between H_2_S/CO-producing systems, where CO generation by HO-2 inhibits astrocytic CBS together with subsequent release of vasodilatory H_2_S. During hypoxia, HO-2 activity is blocked by the limited oxygen supply that allows CBS to generate H_2_S, leading to vasodilation [159]. In the liver, bile excretion is also regulated by CBS in a CO-dependent manner [160]. Besides, inhibition of CBS activity by CO alters redox homeostasis in breast cancer, improving its sensitivity to chemotherapeutics [161] (Figure 5).

In isolated bovine aorta, the HO inhibitor ZnPP-IX increases endogenous H_2_S production in a concentration-dependent manner, whereas hemin attenuates the basal H_2_S levels [162]. In a rat renal injury model, NaHS improves kidney function, lowers renal nitric oxide (NO) and TNF-α, but increases antioxidant, HO-1 activity, and IL-10 concentration [163]. However, ZnPP-IX damages kidney function. In an aspirin-induced hemorrhagic gastric mucosal model, both CORM-2 and NaHS have a gastroprotective effect; more importantly, the protective effect of CORM-2 seemed to be H_2_S independent [164].

The protective effect of H_2_S in a variety of pathologies might be explained, at least partly, by its HO-1-inducing effect. H_2_S increases HO-1 expression in human kidney cells [165]. Interestingly, H_2_S diminishes oxygen dependence of the HO activity by specifically reacting with the ferric verdoheme intermediate of heme catabolism, and verdoheme is cleaved by hydrolysis independently of oxygen [166]. By this mechanism, HOs might catabolize heme in a hypoxic environment in the presence of H_2_S. Another example of the positive interaction between the heme and H_2_S system is the CBS deficiency, where the lack of H_2_S disrupts the heme biosynthetic pathway and heme transports [167]. More recently, the HO-1/CO/H_2_S has been suggested as a potential therapeutic intervention in Covid 19 [168].

These suggest that the interplay of HO-1/CO/H_2_S axes might become a hot topic shortly.

## 6. Conclusions and Future Perspectives

The incidence of hemolytic and hemorrhagic diseases is still a leading cause of death worldwide. Based on many previous studies, it is apparent that several biological events are involved in hemolytic/hemorrhagic diseases, including LDL oxidation, inflammation, endoplasmic reticulum stress, inhibition of osteoclast formation, and complement activation. This phenomenon supports the concept that hemoglobin and heme stress triggers a wide range of cellular damages.

CO and H_2_S have been shown to exert remarkable protective properties in hemolytic/hemorrhagic human pathologies. Evidence supports the hypothesis that the HO/CO and H_2_S dynamically interact with each other, but these interactions need to be further elucidated to develop future therapeutics and pharmacological agents releasing CO and H_2_S that might be ideal candidates for decreasing hemoglobin/heme-induced damages.

## Data Availability

No new data were created or analyzed in this study.
